# Cold-Resistance Plasticizers Derived from Bio-Based *Trans*-Aconitic Acid with High Performance on Solvent Extraction Resistance and Volatility Resistance

**DOI:** 10.3390/polym18131671

**Published:** 2026-07-06

**Authors:** Yirui Shen, Xiaomei Wang, Yangyang Xiong, Xinmeng He, Pingping Jiang, Guizhen Xing

**Affiliations:** 1 The National and Local Joint Engineering Research Center for Biomanufacturing of Chiral Chemicals, Zhejiang University of Technology, Hangzhou 310014, China; 2 School of Materials and Chemical Engineering, Ningbo University of Technology, Ningbo 315211, China; w1981288153@foxmail.com (X.W.); wenrousudui@foxmail.com (Y.X.); rjdj88@foxmail.com (X.H.); 3 Zhejiang Boxiao Bio-Pharmaceutical Co., Ltd., Hangzhou 311400, China; gzxing@bx-biopharm.com; 4 Key Laboratory of Synthetic and Biological Colloids, Ministry of Education, School of Chemical and Material Engineering, Jiangnan University, Wuxi 214122, China; ppjiang@jiangnan.edu.cn

**Keywords:** *trans*-aconitic acid, bio-based plasticizer, cold resistance, poly(vinyl chloride), migration resistance, volatility resistance

## Abstract

Dioctyl adipate (DOA) and dioctyl sebacate (DOS) are widely used cold-resistance plasticizers; however, their low molecular weight and weak polarity result in poor thermal stability and migration resistance. Here, we report the synthesis and performance of bio-based cold-resistance plasticizers derived from *trans*-aconitic acid with enhanced migration resistance. Tri-*n*-butyl *trans*-aconitate (TBTA), tri-*n*-hexyl *trans*-aconitate (THTA), and tri-*n*-octyl *trans*-aconitate (TOTA) were synthesized via one-step esterification with aliphatic alcohols and applied in poly(vinyl chloride) (PVC). Compared with commercial plasticizers di-(2-ethylhexyl) phthalate (DEHP), tributyl citrate (TBC) and DOA, the synthesized plasticizers demonstrated excellent thermal stability and cold-resistance. After freezing treatment, the $T_{g}$ values of TBTA/PVC (18.99 °C) and THTA/PVC (20.88 °C) were lower than those of DEHP/PVC (22.74 °C). The branched architecture was supposed to strengthen interactions between plasticizers and PVC, improving volatility resistance and solvent extraction resistance. Compared with DOA/PVC at 48 h, TBTA/PVC, THTA/PVC and TOTA/PVC displayed volatility mass loss reduction of ~1.5%, 4% and 7%, respectively. Their extraction mass loss in ethanol decreased by 5–6%, while in petroleum ether, TBTA/PVC and TOTA/PVC dropped by 11.95% and 2.63%, respectively. These bio-based plasticizers are promising alternatives to the poor migration resistance of conventional low-temperature plasticizers.

## 1. Introduction

Plasticizers are indispensable additives in polymer processing, essential for endowing rigid plastic networks with necessary flexibility and processability [1,2,3,4]. Among all plasticizer families, phthalate esters have long dominated the market owing to their excellent plasticizing efficiency, low cost, and broad compatibility with PVC [5,6,7]. Plastic products deployed in low-temperature environments, such as cold-chain packaging films, refrigerator door gaskets, outdoor flexible pipes, and agricultural films, etc., demand specialized cold-resistance plasticizers [8]. Under low-temperature conditions, the industry has relied predominantly on aliphatic dicarboxylate esters, particularly DOA and DOS, which remain the most widely used cold-resistance plasticizers to date [9,10,11,12]. The linear aliphatic backbone of these compounds disrupts PVC chain packing more effectively than aromatic counterparts at subzero temperatures, yielding substantially depressed glass transition temperatures. However, DOA and DOS suffer from inherently poor compatibility with PVC and are employed as auxiliary agents blended alongside phthalate plasticizers [13]. Additionally, due to their low molecular weight and linear molecular structure, aliphatic dibasic acid esters tend to be volatile and prone to migration from plastic products [14]. Therefore, breaking the trade-off between low-temperature flexibility and migration resistance is a formidable challenge, and the industry urgently requires non-toxic, highly efficient alternatives.

*Trans*-aconitic acid [15] is a biomass-derived tricarboxylic acid with high chemical functionality and potential as a sustainable platform chemical for advanced bio-based materials. Its three carboxyl groups enable the construction of tri-ester plasticizers bearing a branched, multi-chain architecture in a single esterification step. This structure is expected to strengthen plasticizer-PVC molecular interactions, suppress diffusion-driven migration, and simultaneously preserve the aliphatic chain flexibility essential for cold-resistance. Moreover, *trans*-aconitic acid possesses a structure similar to that of citric acid. In particular, citrate ester plasticizers are widely utilized as non-toxic and environmentally benign additives, authorized by the U.S. Food and Drug Administration (FDA) for application in medical devices, food packaging, and children’s toys, etc. [16]. Plasticizers derived from *trans*-aconitic acid similarly demonstrate commendable safety profiles alongside superior functional performance. Furthermore, aconitic acid was identified in the U.S. Department of Energy screening as one of the 30 promising biomass-derived building-block candidates but was classified as a Tier-2 compound [17]. Traditionally, *trans*-aconitic acid has been obtained mainly in small quantities from sugarcane-processing streams. Subsequently, chemical synthesis routes were developed. However, these routes were constrained by low yields, complex procedures, and excessive byproduct generation. Fortunately, rapid progress in biosynthetic approaches has enabled the industrial-scale manufacture of *trans*-aconitic acid. Recently, microbial fermentation has become a more viable production strategy, with engineered Aspergillus terreus enabling green and efficient biosynthesis of *trans*-aconitic acid at titers up to 60 g/L and supporting the establishment of a 100-ton-scale production line [18]. The literature pertaining to the rational design of plasticizers utilizing *trans*-aconitic acid as a precursor feedstock remains notably scarce. Siong et al. [19] synthesized a series of novel aconitate esters via the esterification of aconitic acid with naturally derived alcohols. Although they systematically evaluated the experimental parameters and demonstrated that catalyst selection profoundly dictated selectivity, they did not assess the viability of these compounds for use as plasticizers. Hou et al. [20] achieved the green, large-scale production of *trans*-aconitic acid through synthetic biology and microbial fermentation, subsequently employing chemical esterification to react *trans*-aconitic acid with a diverse range of alcohols, yielding *trans*-aconitate esters. The plasticizing performance of these derivatives was systematically evaluated and their applicability was extended across multiple application scenarios. However, the low-temperature performance of these plasticizers was not specifically investigated. Saeed et al. [21] synthesized a hyperbranched polyester utilizing *trans*-aconitic acid and phloroglucinol. Although this polymer exhibited excellent solubility across a diverse array of solvents, its potential application as a plasticizer in polymeric materials was not explored.

In this work, *trans*-aconitate plasticizers were designed and synthesized using *trans*-aconitic acid and linear monohydric alcohol as the starting materials. The cold-resistance of the obtained plasticizers was systematically investigated and compared with DOA, DEHP, and TBC. This study aims to evaluate the low-temperature performance of *trans*-aconitate plasticizers and alleviate the current shortage of high-performance alternatives to conventional low-temperature plasticizers. Owing to its unique tree carboxyl structure, *trans*-aconitic acid can increase the branching degree of plasticizers, thereby contributing to the enhancement of their migration resistance. Moreover, the design and synthesis of *trans*-aconite ester plasticizers from bio-based materials offer safety and environmental benefits, aligning with market demands for high-end, innovative plasticizers. These findings establish *trans*-aconitate esters as a promising bio-based platform for next-generation cold-resistance plasticizers, simultaneously addressing the toxicity concerns of phthalates and the migration deficiencies of current aliphatic plasticizers.

## 2. Materials and Methods

### 2.1. Materials

*Trans*-aconitic acid (AR, ≥98%), *p*-toluene sulfonic acid (*p*-TSA, AR, 99%), and Di-(2-ethylhexyl) phthalate (DEHP, AR, 95%) were purchased from Shanghai Macklin Biochemical Co., Ltd. (Shanghai, China). *n*-butanol(AR), *n*-hexanol (AR), *n*-octanol(AR), cyclohexane (AR), sodium hydroxide (AR), phenolphthalein (AR), sodium chloride (AR), tetrahydrofuran (THF, AR), anhydrous magnesium sulfate (AR), petroleum ether (AR), acetic acid (AR), ethanol (AR), *n*-hexane (AR), and activated carbon (AR) were obtained from Sinopharm Chemical Reagent Co., Ltd. (Shanghai, China). PVC resin powder (HG-1300, no additives) was purchased from Hanwha Chemical (Ningbo, China) Co., Ltd. Tributyl citrate (TBC, 99%) and dioctyl adipate (DOA, 98%) were provided by Bluesail Chemicals Co., Ltd. (Zibo, China). All water used in the experiments was deionized water.

### 2.2. Synthesis of Trans-Aconitate Plasticizer

#### 2.2.1. Synthesis of Tri-*n*-Butyl *Trans*-Aconitate (TBTA)

The synthetic route was referenced from the literature [19]. An amount of 17.41 g of *trans*-aconitic acid (0.1 mol), 23.72 g of *n*-butanol (0.32 mol), 0.82 g of *p*-toluene sulfonic acid catalyst (2% of the total reactant mass), and 5 mL of cyclohexane as an azeotropic solvent were added to a three-necked flask equipped with a water separator and a spherical condenser. An appropriate amount of cyclohexane was introduced into the water separator, and magnetic stirring was initiated. The system was purged with nitrogen for 20 min to ensure an inert atmosphere. The reaction temperature was maintained at 100–120 °C, and the reaction duration was monitored. The progress of the reaction was tracked by measuring the change in acid value and observing the accumulation of water in the water separator. The reaction duration was about 1–2 h. Upon completion of the reaction, the product was washed with saturated brine until neutral pH, and the organic layer was separated for vacuum distillation. The distilled product was further dried over anhydrous magnesium sulfate. The resulting plasticizer was identified as tri-*n*-butyl *trans*-aconitate (TBTA) and the final isolated yield was calculated to be around 80.31%.

#### 2.2.2. Synthesis of Tri-*n*-Hexyl *Trans*-Aconitate (THTA)

An amount of 17.41 g of *trans*-aconitic acid (0.1 mol), 32.69 g of *n*-hexanol (0.32 mol), 1.00 g of *p*-toluene sulfonic acid catalyst (2% of the total reactant mass), and 5 mL of cyclohexane as an azeotropic solvent were added to a three-necked flask equipped with a water separator and a spherical condenser. An appropriate amount of cyclohexane was introduced into the water separator, and magnetic stirring was initiated. The system was purged with nitrogen for 20 min to ensure an inert atmosphere. The reaction temperature was maintained at 120–140 °C, and the reaction duration was monitored. The progress of the reaction was tracked by measuring the change in acid value and observing the accumulation of water in the water separator. The reaction duration was about 2–3 h. Upon completion of the reaction, the product was washed with saturated brine until neutral pH, and the organic layer was separated for vacuum distillation. The distilled product was further dried over anhydrous magnesium sulfate. The resulting plasticizer was identified as tri-*n*-hexyl *trans*-aconitate (THTA) and the final isolated yield was calculated to be around 81.39%.

#### 2.2.3. Synthesis of Tri-*n*-Octyl *Trans*-Aconitate (TOTA)

**Scheme 1:**
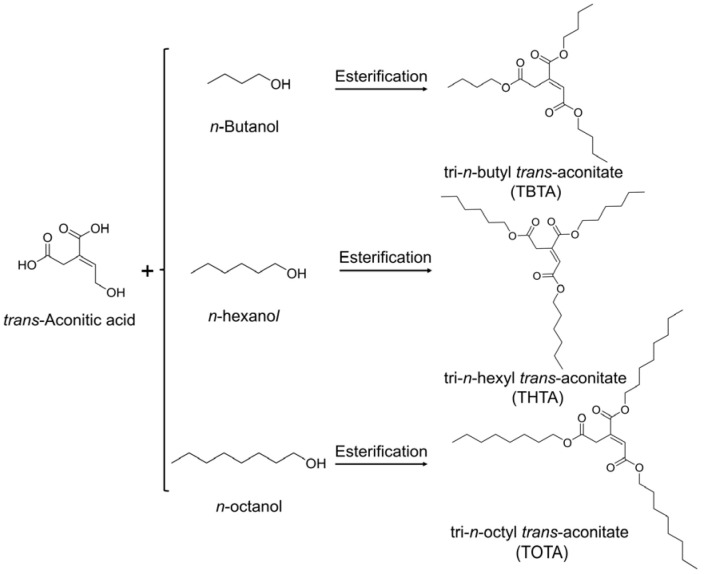
Synthetic route of TBTA, THTA and TOTA.

An amount of 17.41 g of *trans*-aconitic acid (0.1 mol), 41.67 g of *n*-octanol (0.32 mol), 1.18 g of *p*-toluene sulfonic acid catalyst (2% of the total reactant mass), and 5 mL of cyclohexane as an azeotropic solvent were added to a three-necked flask equipped with a water separator and a spherical condenser. An appropriate amount of cyclohexane was introduced into the water separator, and magnetic stirring was initiated. The system was purged with nitrogen for 20 min to ensure an inert atmosphere. The reaction temperature was maintained at 140–180 °C, and the reaction duration was monitored. The progress of the reaction was tracked by measuring the change in acid value and observing the accumulation of water in the water separator. The reaction duration was about 1.5–2 h. Upon completion of the reaction, the product was washed with saturated brine until neutral pH, and the organic layer was separated for vacuum distillation. The distilled product was further dried over anhydrous magnesium sulfate. The resulting plasticizer was identified as tri-*n*-octyl *trans*-aconitate (TOTA) and the final isolated yield was calculated to be around 85.34%. The synthetic route of TBTA, THTA and TOTA is illustrated in Scheme 1. The reactant ratio was designed to ensure complete reaction of *trans*-aconitic acid. Although excess alcohol was used, the excess proportion was kept low because *n*-hexanol and *n*-octanol were not easy to remove.

### 2.3. Preparation of PVC Specimens

The three plasticizers obtained were applied to PVC resin and the performance of PVC specimens was evaluated. PVC specimens were fabricated through the solvent casting method [22]. In detail, 12 g of PVC resin powder, 6 g of plasticizer (50% based on resin mass) and 150 mL of THF were mixed in a 250 mL beaker with constant magnetic stirring for one night. During the stirring process, the beaker was sealed with plastic wrap in order to prevent the fast volatilization of THF. When the mixture became absolutely transparent, it was poured into a Petri dish of 15 cm diameter. Afterwards, the mixture was evaporated at ambient temperature for 3–5 days and further dried at 40 °C for another 2–3 days. The PVC sample was obtained after the solvent was absolutely evaporated and denoted as TBTA/PVC, TOTA/PVC, and THTA/PVC, respectively. Similarly, DOA/PVC, DEHP/PVC and TBC/PVC were prepared using the aforementioned method.

### 2.4. Characterization Methods

The acid value changes during the synthesis of plasticizers were determined according to the Chinese standard GB/T 1668-2008 [23]. To minimize experimental error, each measurement was performed in triplicate, with the arithmetic mean reported as the definitive acid value. The molecular structures of the synthesized plasticizers were characterized using a Fourier transform infrared (FT-IR) spectrometer (Nicolet iS50, Thermo Fisher Scientific, Waltham, MA, USA). The number of scans was set to 32, the resolution was set to 4 cm^−1^, and the scanning range was set to 500–4000 cm^−1^. The structural integrity and purity of the synthesized plasticizers were further corroborated by ^1^H nuclear magnetic resonance (^1^H NMR) spectroscopy (500 Ascend, Ascend Performance Materials, Houston, TX, USA), with deuterated chloroform (CDCl_3_) employed as the solvent. Gas Chromatography–Mass Spectrometry (GC-MS, Agilent 7890A-5975C, Agilent, Santa Clara, CA, USA) was used to analyze the purity of the as-synthesized plasticizers. The chromatographic separation was performed on an Agilent HP-5 capillary column (30 m × 0.25 mm × 0.25 μm).

Shore A hardness measurements were performed using a Shore A durometer (LX-A, Shanghai Wanheng Precision Instrument Factory, Shanghai, China). PVC film specimens were cut into 6 cm × 6 cm squares and placed flat on a smooth glass plate, after which five hardness readings were recorded at distinct positions across each specimen; the arithmetic mean of these five measurements was reported as the final hardness value. Thermogravimetric analysis (TGA) was performed on a thermogravimetric analyzer (STA 2500 Regulus, Netzsch, Selb, Germany). Approximately 8–10 mg of each PVC specimen was heated from 50 °C to 600 °C at a rate of 20 °C/min under a nitrogen atmosphere maintained at a flow rate of 20 mL/min to assess thermal stability. The glass transition temperature (*T_g_*) of the PVC specimens was determined by dynamic mechanical analysis (DMA) using a DMA8000 analyzer (PerkinElmer, Shelton, CT, USA). The measurements were conducted over a temperature range of −80°C to 150°C at a heating rate of 3°C/min and an oscillation frequency of 1 Hz under a nitrogen atmosphere. The sample dimensions were set to 30 mm × 6 mm × 1 mm. Tensile strength and elongation at break were evaluated to assess the plasticizing efficacy of the synthesized compounds, with all mechanical tests performed in accordance with ISO 527-5:2021 [24] using a servo-controlled universal testing machine (AI-7000-LA10, Gotech Testing Machines, Dongguan, China). Dumbbell-shaped specimens were precision-cut from PVC sheets using a custom mold, with a gauge length of 10 mm, a width of 2 mm, and a thickness of 1 mm. A crosshead speed of 50 mm/min was applied throughout testing, and each reported value represents the mean of three independent measurements.

The volatility resistance of the PVC specimens was evaluated in accordance with ISO 176-2005 [25]. Specimens were cut into square pieces measuring 30 mm × 30 mm × 1 mm and subsequently dried in a desiccator for 6 h, after which their initial masses were precisely recorded to the nearest 0.0001 g as $m_{0}$. A 50 mL ceramic crucible was charged with an appropriate quantity of activated carbon powder, and each pre-weighed specimen was fully embedded within the activated carbon. The crucible assembly was then placed in a constant-temperature oven maintained at 100 °C. At predetermined time intervals, specimens were retrieved, residual activated carbon was carefully removed from the surface using filter paper, and the mass was accurately reweighed and recorded as m. Each measurement was conducted in triplicate, with the mean value reported as the final result. Volatility resistance was quantified by the volatility mass loss rate, calculated according to Equation (1).

(1) $$\eta_{1}=\frac{m_{0}-m}{m_{0}}\times 100$$

where *η*_1_ represents the volatility mass loss rate, with the unit of %; *m*_0_ denotes the mass of the PVC sheet before the test, with the unit of g; and *m* denotes the mass of the PVC sheet after the test, with the unit of g.

The extraction resistance of the PVC specimens was measured in accordance with ISO 175-2010 [26], using distilled water, a polar solvent (absolute ethanol), and a non-polar solvent (petroleum ether) as immersion media. Specimens were cut into 30 mm × 30 mm × 1 mm squares and dried in a desiccator for 6 h, after which their initial masses were precisely recorded as *w*_0_ using an analytical balance. The specimens were subsequently immersed in 50 mL ground-stoppered conical flasks containing equal volumes of the respective solvents and maintained in a constant-temperature incubator at 30 °C. At predetermined time intervals, specimens were retrieved, surface solvent was carefully removed with filter paper, and the samples were dried in an oven at 40 °C. Upon cooling to room temperature, the masses were reweighed and recorded as *w*. To ensure statistical reliability, extraction mass loss rates were determined for three parallel specimens from each PVC sample, with the arithmetic mean reported as the final result. The extraction mass loss rate was calculated according to Equation (2).

(2) $$\eta_{2}=\frac{w_{0}-w}{w_{0}}\times 100$$

Here, *η*_2_ represents the extraction mass loss rate, with the unit of %; *w*_0_ denotes the mass of the PVC sheet before immersion, with the unit of g; and *w* denotes the mass of the PVC sheet after immersion, with the unit of g.

## 3. Results and Discussion

### 3.1. Characterization of Plasticizers

#### 3.1.1. FT-IR Spectra of TBTA, THTA and TOTA Plasticizers

**Figure 1:**
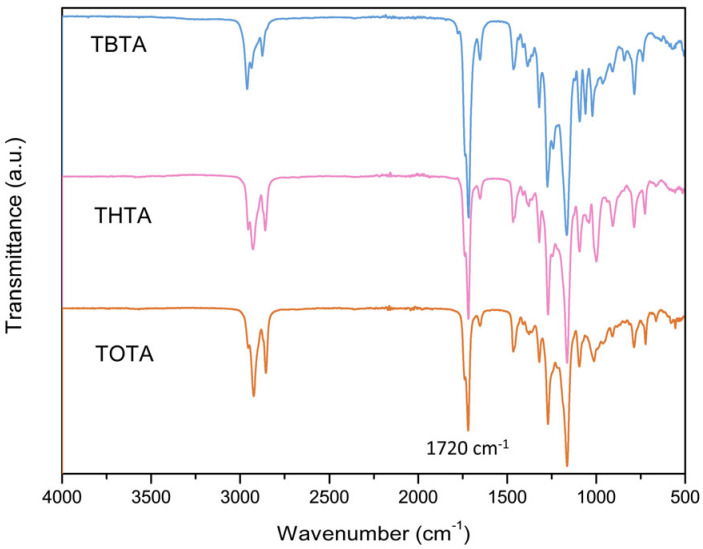
FT-IR spectra of TBTA, THTA and TOTA.

The FT-IR spectra of TBTA, THTA and TOTA are shown in Figure 1. As observed from the spectra, no broad absorption band corresponding to hydroxyl groups (−OH) was detected around 3500 cm^−1^ [27,28]. Meanwhile, the characteristic stretching vibration peak of C=O appeared at approximately 1720 cm^−1^, confirming the formation of ester products [29,30].

#### 3.1.2. ^1^H NMR Spectra of TBTA, THTA and TOTA Plasticizers

**Figure 2:**
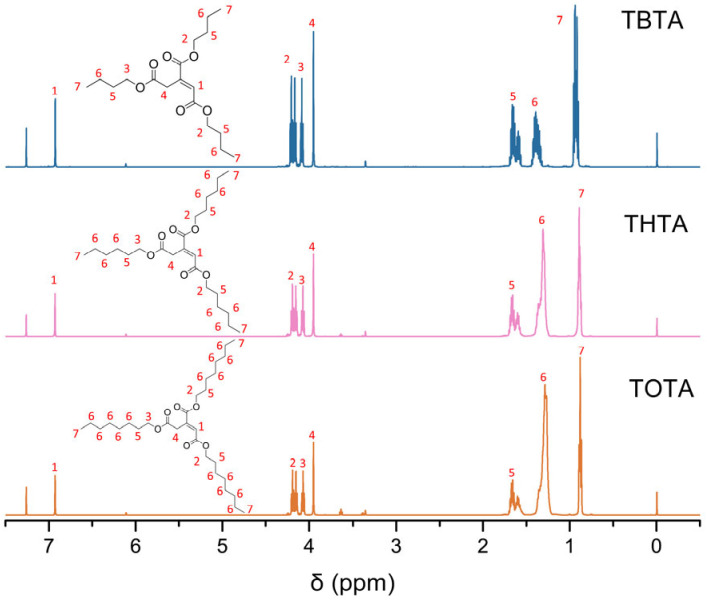
^1^H NMR spectra of TBTA, THTA and TOTA.

The ^1^H NMR spectra of TBTA, THTA and TOTA are shown in Figure 2, and the structure of all the plasticizers matched well with the corresponding ^1^H NMR data. The NMR spectral data are in good agreement with the literature precedents [31]. The detailed ^1^H NMR data were listed, respectively. TBTA: ^1^H NMR (500 MHz, CDCl_3_) δ:6.93 (s, 1 H), 4.22~4.15 (m, 4 H), 4.10~4.07 (t, J = 4 Hz, 2 H), 3.95 (s, 2 H), 1.68~1.58 (m, 6 H), 1.42~1.33 (m, 6 H), 0.96~0.90 (m, 9 H); THTA: ^1^H NMR (500 MHz, CDCl_3_) δ:6.93 (s, 1 H), 4.21~4.14 (m, 4 H), 4.08~4.06 (t, J = 4 Hz, 2 H), 3.95 (s, 2 H), 1.69~1.59 (m, 6 H), 1.38~1.30 (m, 18 H), 0.90~0.87 (m, 9 H); and TOTA: ^1^H NMR (500 MHz, CDCl_3_) δ 6.93 (s, 1 H), 4.20~4.14 (m, 4 H), 4.08~4.06 (t, J = 4 Hz, 2 H), 3.95 (s, 2H), 1.69~1.59 (m, 6 H), 1.36~1.27 (m, 30 H), and 0.89~0.87 (m, 9H). GC-MS analysis was performed to verify the purity of TBTA and THTA, and the corresponding total ion chromatograms are shown in Figures S1 and S2. The total ion chromatograms (TICs) of TBTA and THTA each displayed a single sharp peak, demonstrating the high purity of the synthesized plasticizers. No chromatographic peak of TOTA was observed in GC-MS analysis because of its extremely high boiling point.

### 3.2. The Application Performance of Plasticizers in PVC

#### 3.2.1. Morphological and Hardness Variations in PVC Specimens Before and After Freezing

The synthesized plasticizers TBTA, THTA, and TOTA were applied in PVC resin powder, and the PVC specimens were designated as TBTA/PVC, THTA/PVC, and TOTA/PVC, respectively. To evaluate the low-temperature performance of these plasticizers, the PVC specimens were subjected to freezing at −20°C for 30 days. The properties of PVC specimens were compared before and after low-temperature treatment. The appearance and the hardness data of all PVC specimens are summarized in Table 1. As evidenced by the table, no significant alterations in surface appearance were observed in any of the plasticized PVC specimens after low-temperature treatment. Further quantitative assessment via Shore A hardness measurements revealed a moderate increase in hardness after freezing. However, the magnitude of hardness variation in TBTA/PVC, THTA/PVC, and TOTA/PVC remained comparable to that of PVC plasticized with the benchmark low-temperature plasticizer DOA. These results demonstrated that the synthesized plasticizers impart excellent low-temperature resistance to PVC, with negligible deterioration in both appearance and mechanical hardness upon prolonged cold-temperature exposure. DEHP is a well-recognized plasticizer with comprehensive properties and excellent low-temperature performance [32]. After long-term treatment in a low-temperature environment, its hardness did not increase but decreased slightly. This change was negligible, which was within the range of measurement error. Moreover, UV-visible transmittance analysis could provide valuable information for evaluating the optical properties of the samples; this characterization was not included in the present study due to the limitation of available experimental facilities. This study aimed to explore the changes in PVC performances before and after freezing. Obvious visual evidence indicated no alteration in the morphology and flexibility of PVC samples. The results have provided sufficient evidence to support the main conclusions. Future work will include systematic UV-visible transmittance measurements to further clarify the optical performance of these materials.

#### 3.2.2. Mechanical Properties

**Figure 3:**
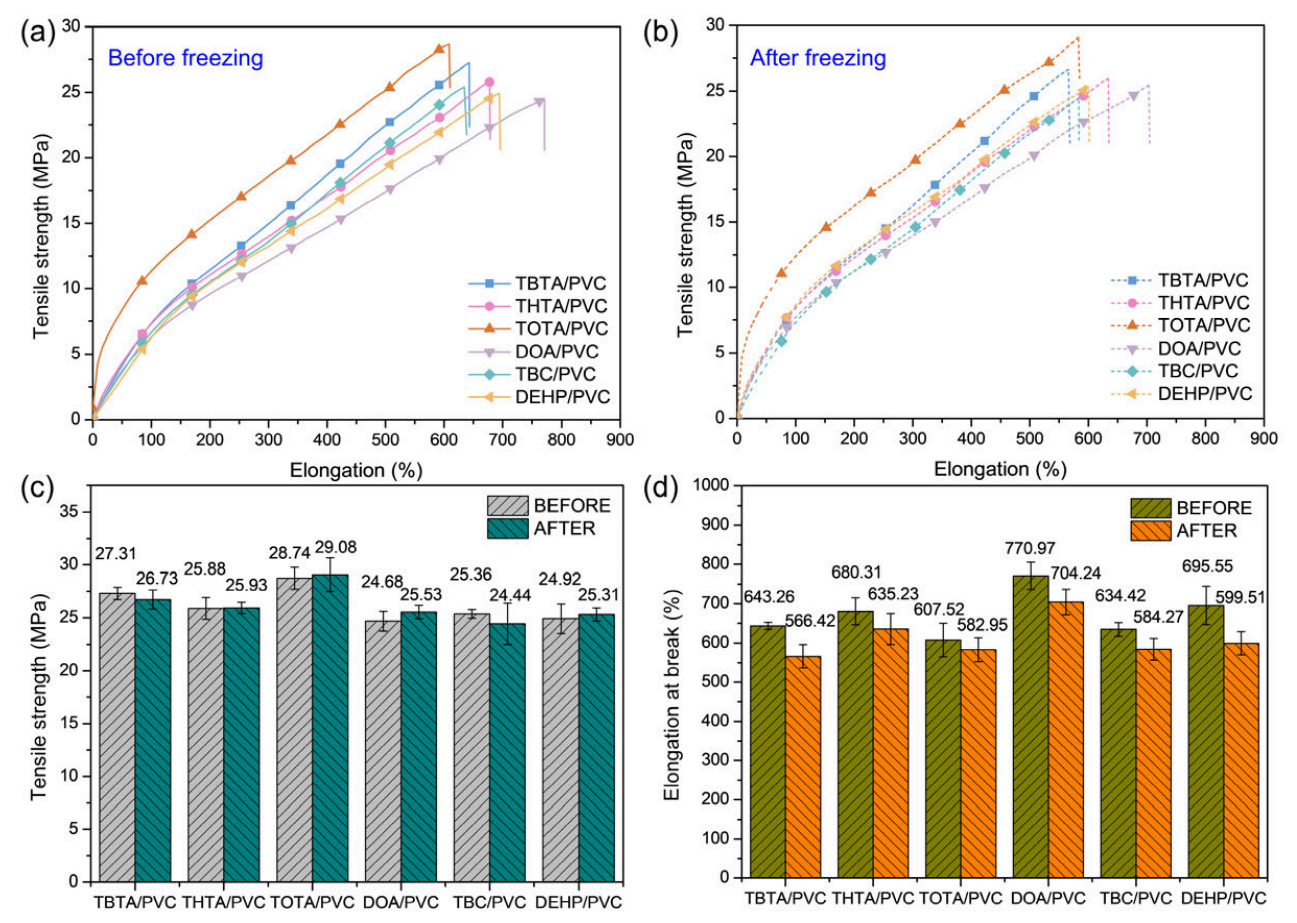
(**a**) Tensile stress–strain curves of PVC specimens before freezing; (**b**) tensile stress–strain curves of PVC specimens after freezing; (**c**) tensile strength and elongation at break data of PVC specimens before freezing; (**d**) tensile strength and elongation at break data of PVC specimens after freezing.

The mechanical properties of the PVC specimens are presented in Figure 3a–d. Tensile strengths of the specimens ranked in the order as follows: TOTA/PVC > TBTA/PVC > THTA/PVC > TBC/PVC > DEHP/PVC > DOA/PVC. Elongation at break followed the sequence: DOA/PVC > DEHP/PVC > THTA/PVC > TBTA/PVC > TBC/PVC > TOTA/PVC. Overall, THTA/PVC demonstrated the most favorable balance between tensile strength and elongation at break. The tensile strengths of TOTA/PVC, TBTA/PVC and THTA/PVC were superior to those of TBC/PVC, DEHP/PVC and DOA/PVC both before and after freezing. This can be attributed to the presence of three ester groups in the aconitate-based plasticizer. It was proposed that the structure with three ester groups conferred a high degree of structural branching and facilitated its binding with PVC molecules [33]. This branched structure resulted in the increased rigidity of the PVC specimens while maintaining satisfactory tensile strength [34]. Furthermore, it was believed that the alkyl chains derived from linear alcohols provided sufficient flexibility to the PVC matrix [35]. We speculated that the synergistic effect of the above two structural units achieved a favorable balance between tensile strength and elongation. Comparing the specific tensile strength before and after freezing, TBTA/PVC and TBC/PVC exhibited reductions by 2.12% and 3.63%, respectively. Except for TBTA/PVC and TBC/PVC, the tensile strength of other specimens slightly increased due to the reduced flexibility of the PVC specimens. Specifically, after freezing, the tensile strength of THTA/PVC, TOTA/PVC, DOA/PVC, and DEHP/PVC was observed to increase by 0.19%, 1.18%, 3.44%, and 1.57%, respectively. Conversely, elongation at break decreased across all specimens after prolonged freezing: TBTA/PVC, THTA/PVC, TOTA/PVC, DOA/PVC, TBC/PVC, and DEHP/PVC exhibited reductions of 11.95%, 6.63%, 4.04%, 8.66%, 7.90%, and 13.81%, respectively. Notably, the reductions observed for THTA/PVC and TOTA/PVC were smaller than those of the commercial plasticizers DOA/PVC, TBC/PVC, and DEHP/PVC. Furthermore, the reduction for TBTA/PVC also remained inferior to that of DEHP/PVC. Elongation at break after freezing followed the sequence: DOA/PVC > THTA/PVC > DEHP/PVC > TOTA/PVC ≈ TBC/PVC > TBTA/PVC. These findings indicated that aconitate ester plasticizers effectively retained favorable mechanical properties under low temperature conditions, suggesting promising applicability in cold environment applications such as food wrap films and refrigerator sealing strips.

#### 3.2.3. TGA

**Figure 4:**
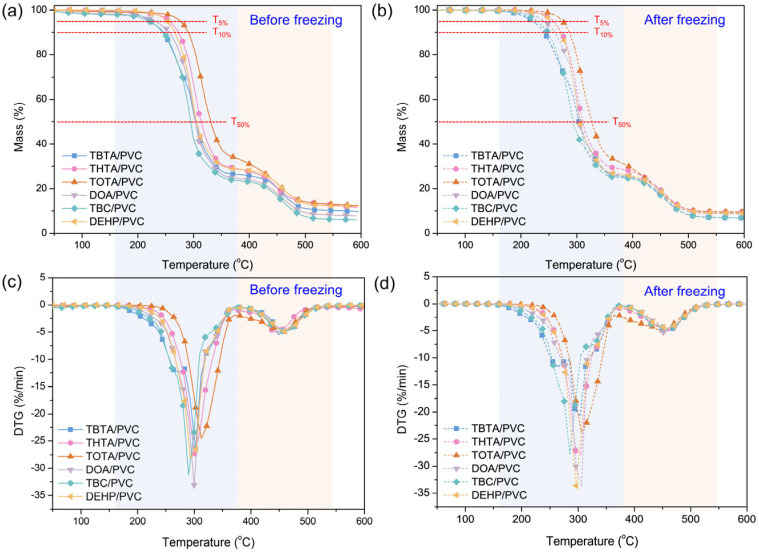
TG curves of PVC specimens (**a**) before and (**b**) after freezing; DTG curves of PVC specimens (**c**) before and (**d**) after freezing.

The thermal stability of the PVC specimens before and after freezing was evaluated by thermogravimetric analysis (TGA), with the corresponding results presented in Figure 4 and Table 2. As illustrated by the TGA curves in Figure 4a, the TOTA/PVC and THTA/PVC curves were positioned above those of DOA/PVC, TBC/PVC, and DEHP/PVC. Compared with TOTA/PVC and THTA/PVC, TBTA exhibited weaker thermal stability owing to its lower molecular weight. However, the TBTA/PVC curve still closely overlapped with that of the structurally analogous TBC/PVC. The thermal stability of the PVC specimens gradually improved with increasing molecular size and molecular weight [36]. Due to the relatively low molecular weights, TBC (with a molecular weight of 360.44) and DOA (with a molecular weight of 370.57) exhibited higher volatility. The results demonstrated that judicious regulation of molecular structure can significantly enhance thermal stability. In addition, it was also suggested that the use of linear alcohols instead of branched structures as synthetic reactants contributed to improved thermal stability as well. The thermal degradation process proceeded through two distinct stages [37,38,39]. The first stage, occurring at 160–390 °C, was primarily associated with plasticizer decomposition and the initial dehydrochlorination of PVC chains. The second stage, occurring at 390–550 °C, mainly involved structural rearrangement of PVC macromolecules and cleavage of the carbon backbone. In addition, the characteristic decomposition temperatures $T_{5\%}$, $T_{10\%}$, and $T_{50\%}$ of TBTA/PVC, THTA/PVC and TOTA/PVC exhibited only slight change after freezing, confirming their good low-temperature resistance. The thermal stability of plasticizers was also tested and the results are shown in Figure S3. Apparently, TOTA and THTA exhibited far superior thermal stability compared with commercial plasticizers. Due to the smaller molecular weight of TBTA, its thermal stability was not as outstanding as that of TOTA and THTA. But, TBTA also showed better thermal stability than DOA, and its thermal performance was comparable to DEHP.

#### 3.2.4. Dynamic Thermomechanical Analysis

**Figure 5:**
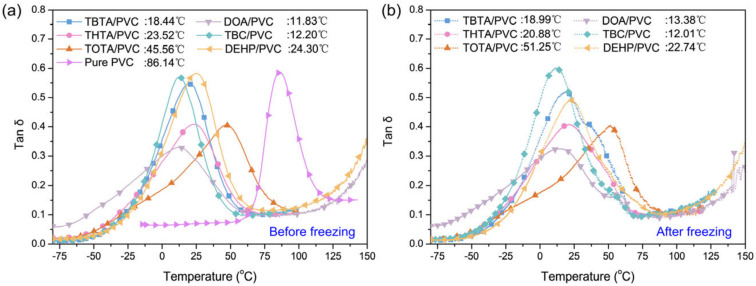
DMA curves of the PVC plasticized by different plasticizers (**a**) before and (**b**) after freezing.

The cold-resistance of PVC specimens can be assessed through their glass transition temperature (*T_g_*). In general, a lower *T_g_* for plasticized PVC indicates greater flexibility at lower temperatures and, consequently, better cold-resistance. The *T_g_* values of different PVC specimens were determined by DMA, and the results are presented in Figure 5. Pure PVC exhibited a relatively high *T_g_* of approximately 86.14 °C due to the restricted mobility of its polymer chains. The incorporation of plasticizers significantly decreases the *T_g_* by increasing the free volume and enhancing the chain segmental mobility. The *T_g_* values of TBTA/PVC and THTA/PVC exhibited negligible variation before and after freezing and remained lower than those of DEHP-plasticized PVC. The results confirmed that TBTA and THTA maintained effective plasticizing efficiency under prolonged low temperature conditions. In contrast, TOTA with a larger molecular structure exhibited a higher *T_g_* and increased further after freezing. The result revealed that the plasticizing efficiency of TOTA at low temperatures was inferior to that of TBTA and THTA. As the molecular weight increased, the alkyl chains appended to the branched side chains became longer. Long alkyl chains contain insufficient polar functionality and therefore cannot effectively interact with the polar moieties of PVC chains [40]. Moreover, it was believed that excessively long alkyl chains promoted strong nonpolar intermolecular interactions among plasticizer molecules, thereby further diminishing plasticization efficiency [41]. At the macroscopic level, this led to enhanced thermal stability, accompanied by an increase in the glass transition temperature.

#### 3.2.5. Analysis of Volatility Resistance

**Figure 6:**
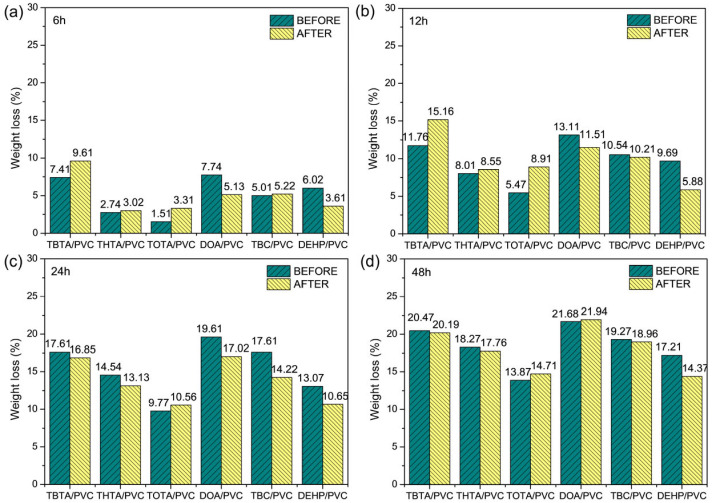
Weight loss of different PVC films after volatility testing, (**a**) 6 h, (**b**) 12 h, (**c**) 24 h and (**d**) 48 h.

Figure 6 presents the volatility resistance of PVC specimens in activated carbon at 100 °C. After 48 h of testing, the volatility mass loss of all PVC specimens showed negligible variation before and after freezing, confirming that all specimens maintained excellent low-temperature resistance and retained their original volatility resistance under prolonged low-temperature conditions. Analysis of the volatility resistance data revealed a progressive enhancement with increasing molecular size of the aconitate ester plasticizers, following the order TOTA/PVC > THTA/PVC > TBTA/PVC. This trend was consistent with the well-acknowledged principle that plasticizers of higher molecular weight possessed superior thermal stability [42]. In addition, the elongated side-chain structures strengthened the interactions with PVC molecular chains, thereby enhancing volatility resistance. Compared to the benchmark commercial low-temperature plasticizer DOA, all three aconitate ester-plasticized PVC specimens demonstrated markedly superior volatility resistance. TBTA/PVC, THTA/PVC and TOTA/PVC reduced volatility mass loss by approximately 1.5–2%, 3.5–4% and 7–8% relative to DOA/PVC, respectively. In comparison with the structurally analogous TBC, TBTA/PVC exhibited comparable volatility resistance to TBC/PVC. However, as the molecular structure increased, both TOTA/PVC and THTA/PVC surpassed TBC/PVC in volatility resistance. The raw alcohol used in both TOTA and DEHP was octanol. Compared with DEHP/PVC, the three-branched molecular structure of TOTA was presumed to promote stronger interactions with PVC chains and consequently provide superior volatility resistance [43]. These findings are in good accordance with the thermogravimetric analysis results.

#### 3.2.6. Analysis of Extraction Resistance

**Figure 7:**
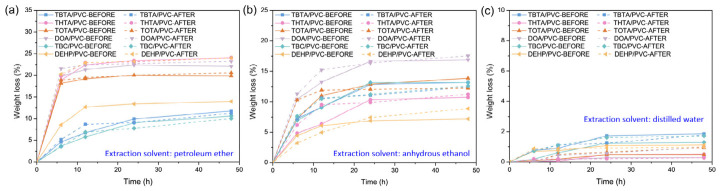
Analysis of the extraction resistance of PVC specimens in (**a**) petroleum ether, (**b**) anhydrous ethanol and (**c**) water.

The extraction resistance of the PVC specimens was evaluated in petroleum ether, anhydrous ethanol, and distilled water, and the results are presented in Figure 7. In the extraction solvent of water, all PVC specimens, both before and after freezing treatment, demonstrated excellent extraction resistance. After 48 h of extraction in water, the weight loss remained below 2% across all specimens, satisfying the relevant safety requirements.

In the polar solvents, anhydrous ethanol, plasticizer migration was observed. The extraction resistance followed the order DEHP/PVC > THTA/PVC > TBTA/PVC ≈ TBC/PVC > TOTA/PVC > DOA/PVC. Additionally, freezing treatment did not significantly affect the extraction resistance of the specimens. The extraction resistance of PVC plasticized with *trans*-aconitate-based plasticizers in ethanol solution was markedly superior to that of DOA/PVC. It was suggested that the branched architecture of *trans*-aconitate plasticizers more effectively enhanced extraction resistance compared with the linear molecular structure of DOA [44,45]. More specifically, the greater number of ester groups inherent to the aconitate framework was considered to be stronger intermolecular interactions with PVC chains, thereby substantially suppressing plasticizer migration from the polymer matrix.

In non-polar solvents such as petroleum ether, the extraction resistance before freezing treatment followed the order TBC/PVC > TBTA/PVC > DEHP/PVC > TOTA/PVC > DOA/PVC > THTA/PVC. After freezing treatment, the extraction resistance followed the order TBC/PVC > TBTA/PVC > TOTA/PVC > DOA/PVC > THTA/PVC ≈ DEHP/PVC. Except for DEHP/PVC, the other PVC specimens showed negligible variation, indicating that these specimens retained satisfactory resistance under prolonged cold-temperature conditions. Both TBTA/PVC and TBC/PVC demonstrated excellent extraction resistance in petroleum ether, which was attributed to the similar structure between TBTA and TBC. In comparison with TBTA/PVC, THTA/PVC and TOTA/PVC with longer alkyl chains exhibited reduced resistance to hydrocarbon solvents, resulting in diminished extraction resistance in petroleum ether. Nevertheless, TOTA/PVC still performed slightly better extraction resistance than DOA/PVC, whereas THTA/PVC exhibited comparable performance to DOA/PVC. It was suggested that excessively long alkyl chains weaken resistance to hydrocarbon solvents [46] and increase the glass transition temperature, which may cause the plasticizer to solidify under low-temperature conditions. Overall, the optimal plasticizer structure was obtained from *trans*-aconitic acid and C6 alcohol.

#### 3.2.7. Analysis of Migration Resistance in Oil-Based Environments

**Figure 8:**
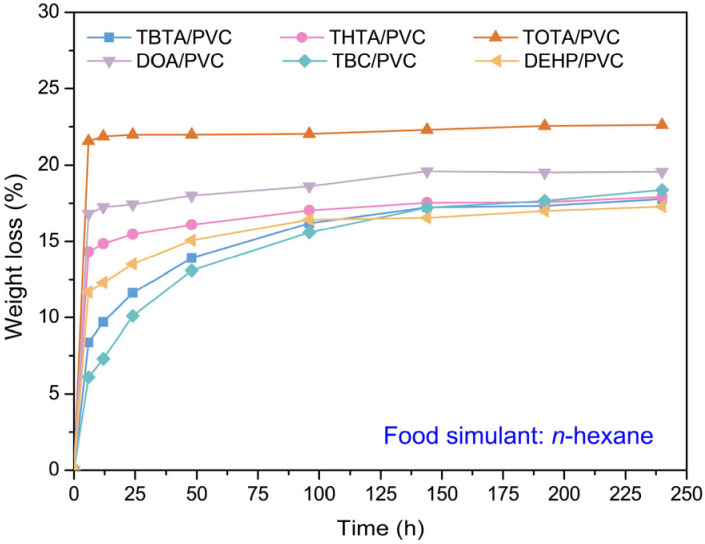
Analysis of the extraction resistance of PVC specimens in *n*-Hexane.

Plasticizers are highly susceptible to migration in oleaginous environments. Utilizing *n*-hexane as a fatty food simulant, the migration resistance of the PVC specimens was evaluated, with the results delineated in Figure 8. The DOA/PVC blend exhibited a substantial extraction loss in *n*-hexane, whereas the migration rates for THTA/PVC and TBTA/PVC were significantly lower, closely approximating those of TBC/PVC and DEHP/PVC. These results demonstrate that THTA and TBTA exhibit superior migration resistance in oil-based environments while retaining excellent low-temperature performance. Unlike the linear aliphatic structure of DOA, THTA and TBTA each contain three functional groups. This branched structure was considered to improve the hydrogen bonding interactions between the carbonyl oxygen atoms and the PVC polymer chains [47], which could effectively restrain the exudation of plasticizer into hydrocarbon solvents.

### 3.3. Plasticization Mechanism

**Figure 9:**
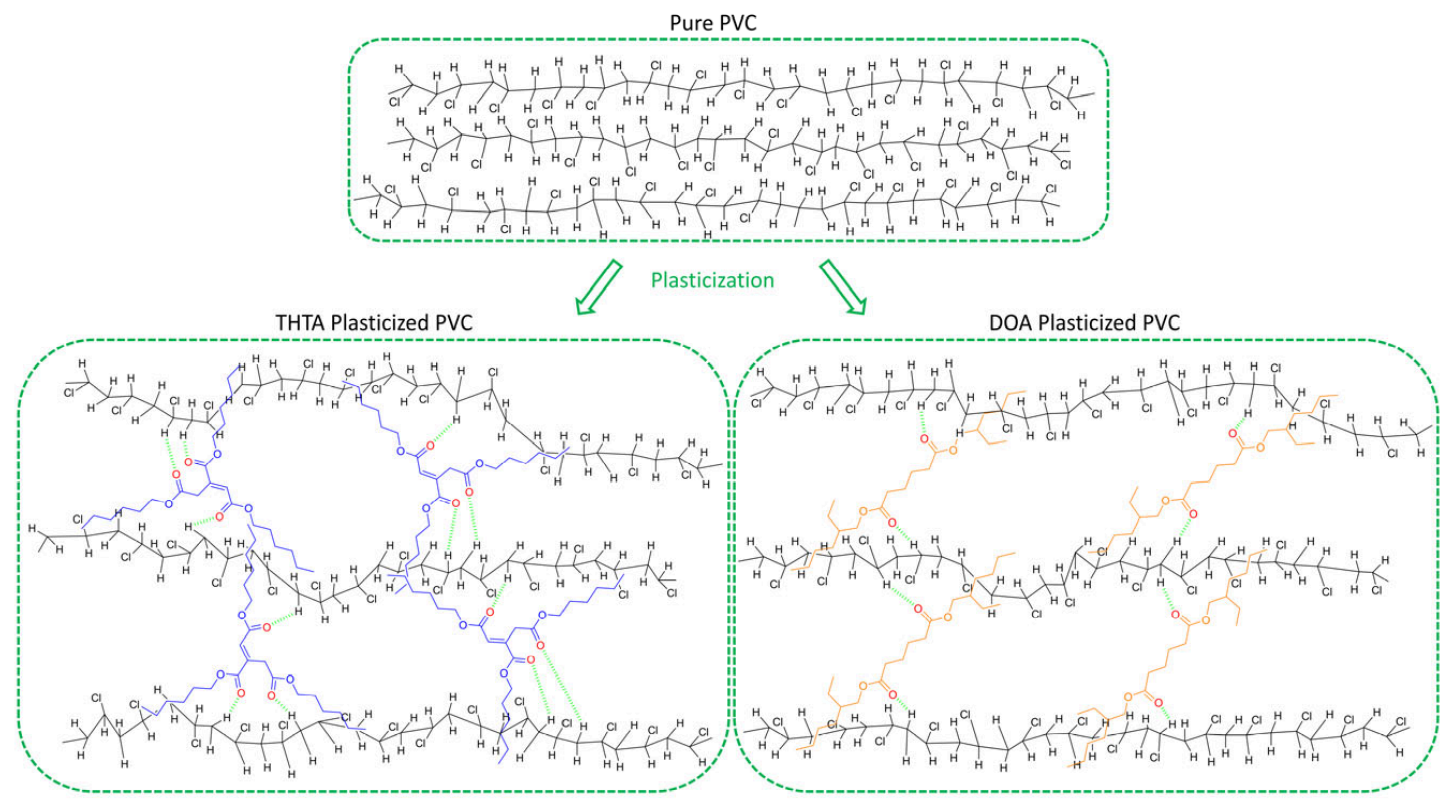
Proposed plasticization mechanism.

As one of the most widely used and effective low-temperature-resistant plasticizers [48], DOA presents two major limitations. First, its relatively low molecular weight (370.57 g/mol) confers high volatility [49]. DOA tends to volatilize during high-temperature processing or service, causing plasticized products to progressively harden and lose flexibility over time. In practical applications, this drawback restricts the allowable loading of DOA in products requiring long-term heat resistance, such as automotive interiors, wires, and cables. Second, although the linear aliphatic structure of DOA imparts excellent low-temperature performance, it also renders DOA susceptible to extraction by hydrocarbon solvents [50]. When plasticized products come into contact with oils or organic solvents, DOA gradually leaches from the PVC matrix, leading to deterioration in material properties. These disadvantages make DOA unsuitable for applications involving oil or solvent exposure. A plausible plasticization mechanism was proposed and presented in Figure 9. Plasticizers designed and synthesized from *trans*-aconitic acid contain three ester groups, which were considered to have stronger interactions with PVC molecular chains. It was suggested that hydrogen-bonding interactions might occur between the oxygen atoms of the C=O groups from plasticizers and the hydrogen atoms of PVC [51]. In addition, the alcohols attached to the ester groups were linear aliphatic alcohols, which were supposed to intertwine and cross-link with PVC chains. This was likely to prevent plasticizer migration from the PVC matrix and improve migration resistance. Meanwhile, it was suggested that the −CH_2_− structure of linear aliphatic alcohols contributed to favorable low-temperature resistance. The interactions between plasticizers and PVC after plasticization were characterized by FT-IR. As shown in Figure S4, a red shift in the carbonyl band was observed after blending PVC with the plasticizer [52].

## 4. Conclusions

Utilizing bio-based *trans*-aconitic acid as the primary feedstock, three *trans*-aconitate plasticizers, tri-*n*-butyl *trans*-aconitate (TBTA), tri-*n*-hexyl *trans*-aconitate (THTA) and tri-*n*-octyl *trans*-aconitate (TOTA), were synthesized via a one-step esterification process employing *n*-butanol, *n*-hexanol and *n*-octanol, respectively. The purity of the products was confirmed by FT-IR, ^1^H NMR and GC-MS. The synthesis of the plasticizers features simple reaction procedures and easily achievable reaction conditions, showing potential for industrial application.

The performance of PVC specimens before and after freezing was systematically analyzed and benchmarked against specimens plasticized with the widely employed commercial plasticizer DEHP, the dedicated low-temperature plasticizer DOA, and the structurally analogous plasticizer TBC. The results demonstrated that the synthesized *trans*-aconitate plasticizers exhibited favorable overall performance in PVC, with only marginal property variations before and after freezing, indicating excellent low-temperature resistance. Specifically, in terms of mechanical properties, THTA/PVC simultaneously exhibited superior elongation at break and tensile strength, with negligible deterioration following freezing treatment. The thermal stability of TOTA/PVC and THTA/PVC surpassed that of DOA/PVC, TBC/PVC and DEHP/PVC. Furthermore, the characteristic decomposition temperatures *T*_5%_, *T*_10%_ and *T*_50%_ remained virtually unchanged before and after freezing. The *T_g_* values of TBTA/PVC and THTA/PVC displayed no appreciable variation after cold-environment treatment and remained lower than that of DEHP/PVC, confirming sustained plasticization efficiency under cold-temperature conditions. The three-branched molecular architecture of *trans*-aconitic acid-derived plasticizers facilitated stronger intermolecular interactions with PVC chains, conferring superior volatility resistance relative to DOA/PVC. Moreover, TBTA/PVC, TOTA/PVC and THTA/PVC exhibited superior extraction resistance in ethanol compared with DOA/PVC. In petroleum ether, TOTA/PVC and TBTA/PVC exhibited substantially greater extraction resistance than DOA/PVC, while THTA/PVC performed comparably. In *n*-hexane, the migration amounts of THTA/PVC and TBTA/PVC were far lower than those of DOA/PVC and approached the levels observed for TBC/PVC and DEHP/PVC.

In summary, THTA, synthesized from the reaction of *trans*-aconitic acid with hexanol, exhibited excellent comprehensive performance. With an appropriate alkyl chain length, it achieved both a low *T_g_* and favorable thermal stability, which effectively compensated for the poor migration resistance of DOA. Compared with the low-temperature-resistant plasticizer DOA, *trans*-aconitate-based plasticizers maintained favorable low-temperature performance (low *T_g_*) while demonstrating improved thermal stability and migration resistance. This greatly broadens their application scope, making them highly promising plasticizer candidates in low-temperature conditions.

## Figures and Tables

**Table 1:** Comparison of different plasticizers plasticized PVC specimens before and after freezing at −20°C for 30 days.

	TBTA/ PVC	THTA/ PVC	TOTA/ PVC	DOA/ PVC	TBC/ PVC	DEHP/ PVC
PVC specimens (before freezing)	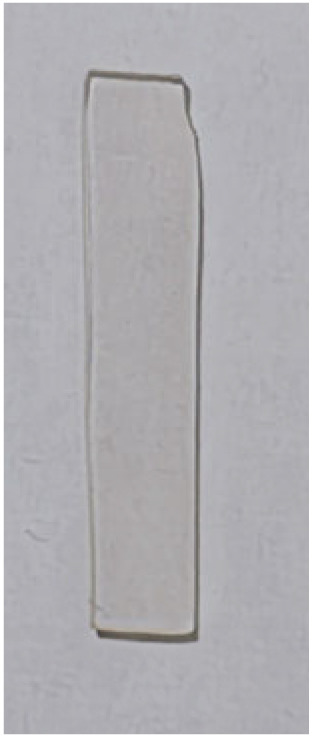	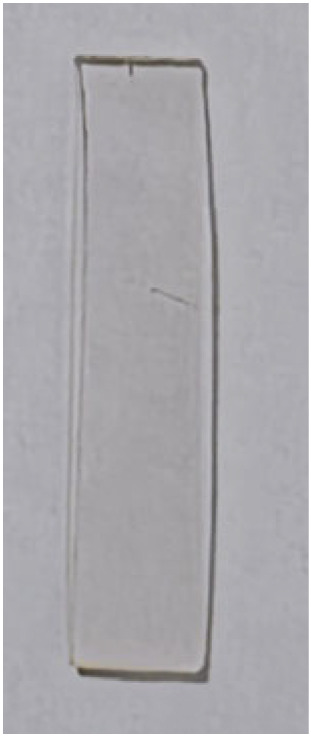	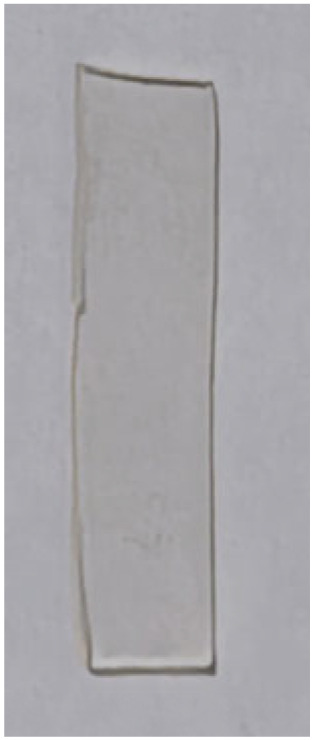	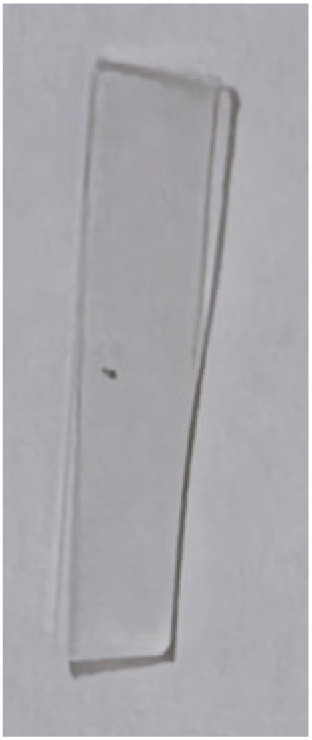	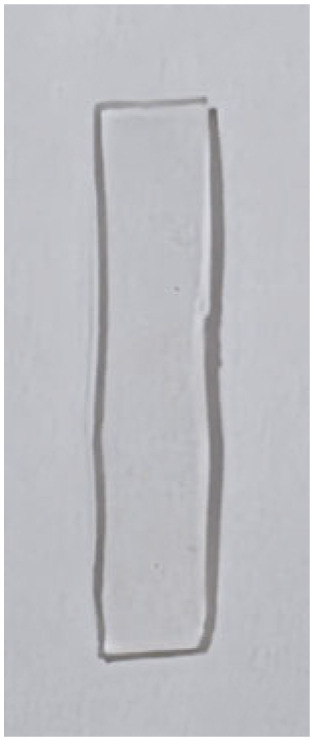	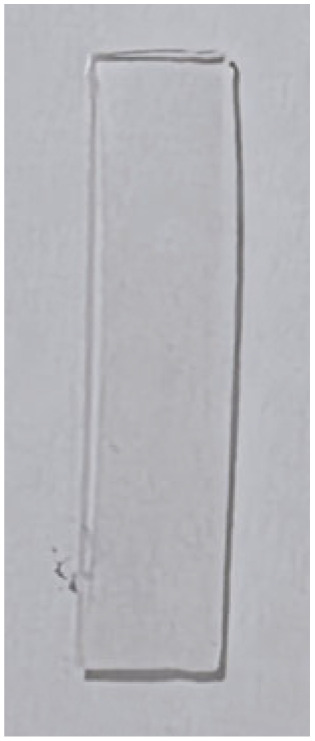
PVC specimens (after freezing)	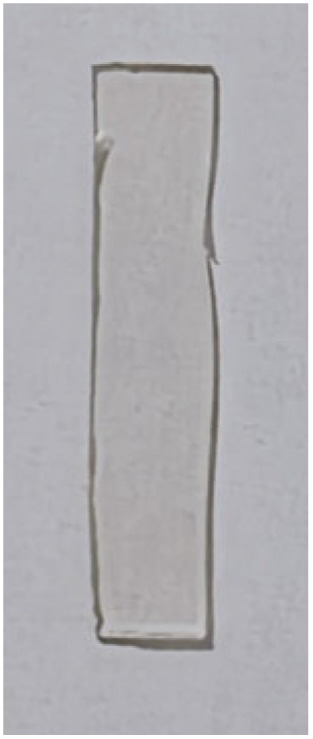	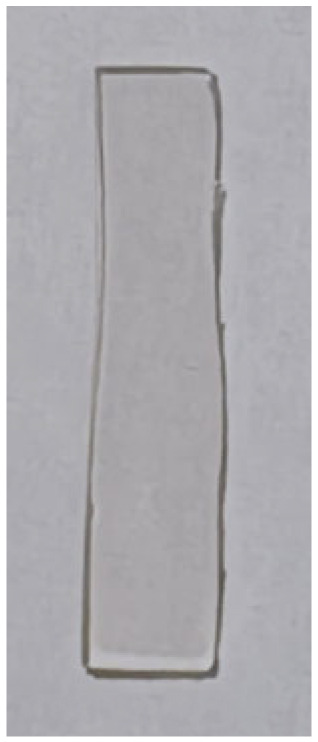	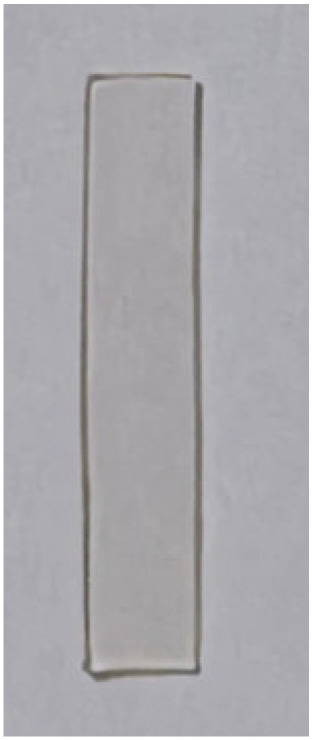	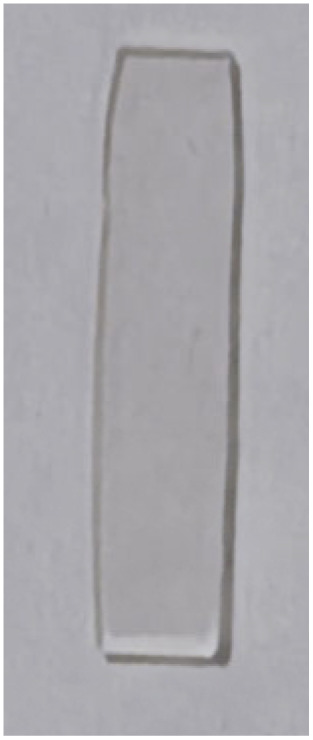	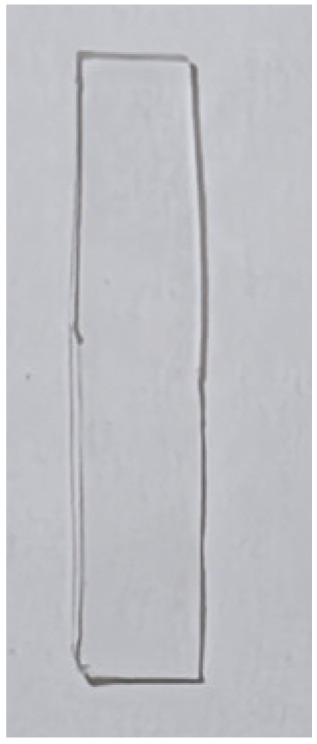	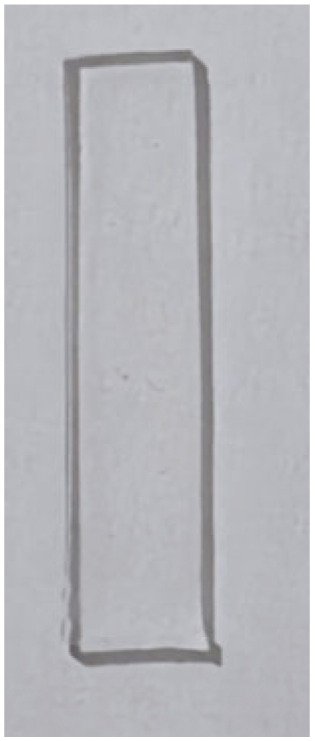
Shore hardness (before freezing, HA)	80.2	76.7	85.3	80.2	84.2	86.3
Shore hardness (after freezing, HA)	87.0	85.2	93.0	87.7	88.8	84.5
Hardness change (%)	+8.48	+11.08	+9.03	+9.35	+5.46	−2.08

**Table 2:** Thermogravimetric data of PVC plasticized by different plasticizers before and after freezing.

PVC Sample	Before Freezing			After Freezing		
	*T*_5%_ (°C)	*T*_10%_ (°C)	*T*_50%_ (°C)	*T*_5%_ (°C)	*T*_10%_ (°C)	*T*_50%_ (°C)
TBTA/PVC	222.61	244.67	303.12	219.82	240.86	303.32
THTA/PVC	257.79	272.88	313.75	258.01	271.81	310.55
TOTA/PVC	276.51	290.34	330.91	273.14	286.48	326.72
DOA/PVC	230.22	256.27	303.69	240.71	259.87	304.77
TBC/PVC	219.37	245.89	295.04	227.83	247.62	293.32
DEHP/PVC	252.18	266.40	305.81	255.22	269.85	305.29

(*T*_5%_, *T*_10%_, and *T*_50%_ denote the temperatures at which PVC specimens undergo 5%, 10%, and 50% mass loss, respectively).

## Data Availability

The original contributions presented in this study are included in the article/Supplementary Materials. Further inquiries can be directed to the corresponding author.

## Supplementary Materials

The following supporting information can be downloaded at: https://www.mdpi.com/article/10.3390/polym18131671/s1, Figure S1: The total ion chromatograms (TICs) of TBTA; Figure S2: The total ion chromatograms (TICs) of THTA; Figure S3: (a) TG and (b) DTG curves of plasticizers; Figure S4: Characteristic carbonyl peaks of plasticizers and plasticized PVC specimens.

## Abbreviations

The following abbreviations are used in this manuscript:

DOA	Dioctyl adipate
DOS	Dioctyl sebacate
TBTA	Tri-*n*-butyl trans-aconate
THTA	Tri-*n*-hexyl *trans*-aconate
TOTA	Tri-*n*-octyl *trans*-aconate
PVC	Poly(vinyl chloride)
DEHP	Di-(2-ethylhexyl) phthalate
TBC	Tributyl citrate
FDA	The U.S. Food and Drug Administration
*p*-TSA	*p*-toluene sulfonic acid
THF	Tetrahydrofuran
FT-IR	Fourier transform infrared
^1^H NMR	^1^H nuclear magnetic resonance
CDCl_3_	Deuterated chloroform
GC-MS	Gas chromatography–mass spectrometry
TIC	Total ion chromatogram
TGA	Thermogravimetric analysis
DMA	Dynamic mechanical analysis
