# Supplementary material for: Cold-Resistance Plasticizers Derived from Bio-Based Trans-Aconitic Acid with High Performance on Solvent Extraction Resistance and Volatility Resistance
Source: Polymers (Basel). 2026 Jul 6;18(13):1671. doi: 10.3390/polym18131671 (PMC13363831; doi:10.3390/polym18131671)
Supplement: Supplementary file 1 [file polymers-18-01671-s001.zip › polymers-4366176-supplementary.pdf]

*Supplementary Materials*

# Cold-Resistance Plasticizers Derived from Bio-Based *Trans*-Aconitic Acid with High Performance on Solvent Extraction Resistance and Volatility Resistance

**Yirui Shen [1,2,3,*], Xiaomei Wang [2], Yangyang Xiong [2], Xinmeng He [2], Pingping Jiang [4] and Guizhen Xing [3]**

[1] The National and Local Joint Engineering Research Center for Biomanufacturing of Chiral Chemicals, Zhejiang University of Technology, Hangzhou 310014, China
[2] School of Materials and Chemical Engineering, Ningbo University of Technology, Ningbo 315211, China; w1981288153@foxmail.com (X.W.); wenrousudui@foxmail.com (Y.X.); rjdj88@foxmail.com (X.H.)
[3] Zhejiang Boxiao Bio-Pharmaceutical Co., Ltd., Hangzhou 311400, China; gzxing@bx-biopharm.com
[4] Key Laboratory of Synthetic and Biological Colloids, Ministry of Education, School of Chemical and Material Engineering, Jiangnan University, Wuxi 214122, China; ppjiang@jiangnan.edu.cn
* Correspondence: shenyr@nbut.edu.cn

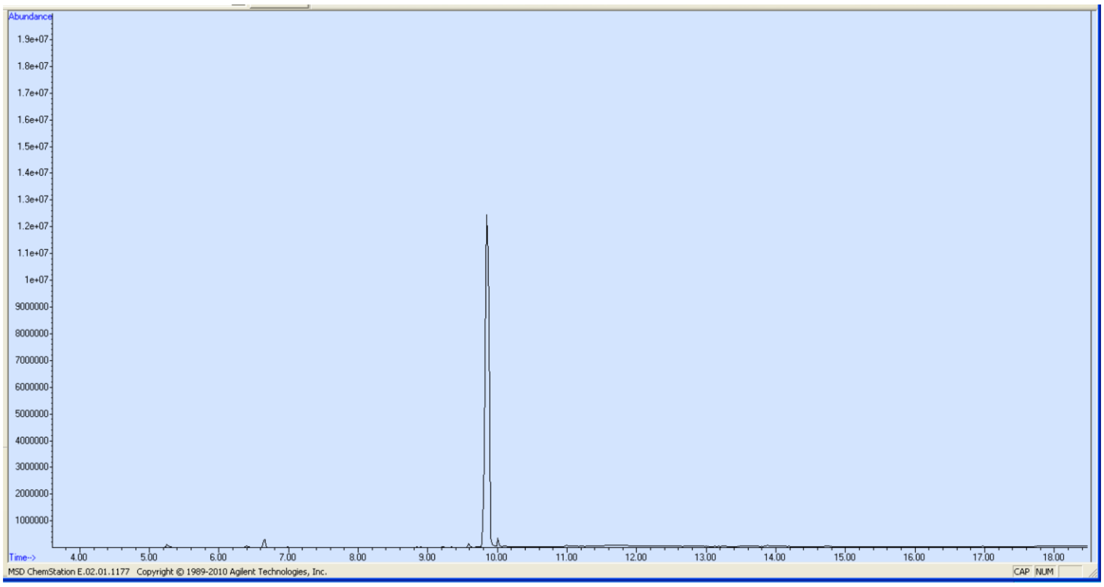


**Figure S1.** The total ion chromatograms (TICs) of TBTA

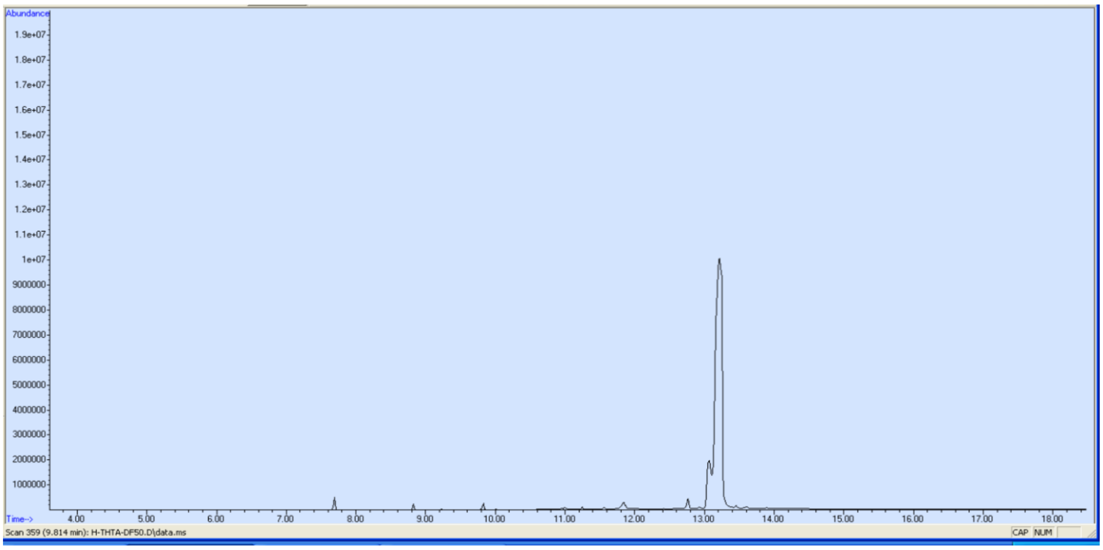


**Figure S2.** The total ion chromatograms (TICs) of THTA

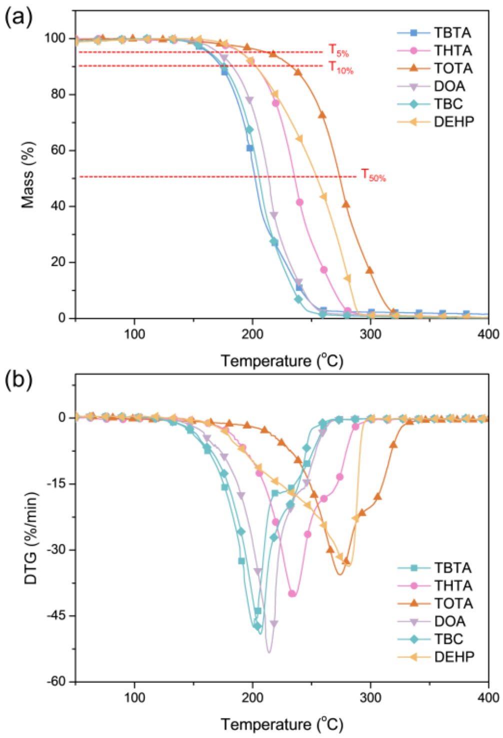


**Figure S3.** (a) TG and (b) DTG curves of plasticizers.

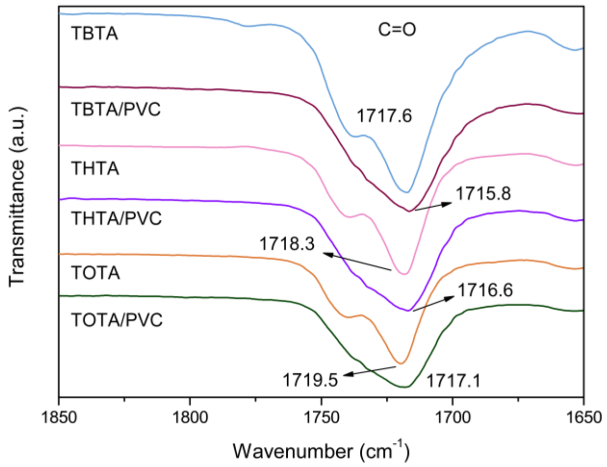


**Figure S4.** Characteristic carbonyl peaks of plasticizers and plasticized PVC specimens.
